# Alcohol-Based Hand Sanitizers: Does Gelling Agent Really Matter?

**DOI:** 10.3390/gels8020087

**Published:** 2022-01-29

**Authors:** Ivana d’Angelo, Romina Provenzano, Ettore Florio, Chiara Pagliuca, Giuseppe Mantova, Elena Scaglione, Mariateresa Vitiello, Roberta Colicchio, Paola Salvatore, Francesca Ungaro, Fabiana Quaglia, Agnese Miro

**Affiliations:** 1 Department of Environmental, Biological and Pharmaceutical Sciences and Technologies, University of Campania “Luigi Vanvitelli”, 81100 Caserta, Italy; ivana.dangelo@unicampania.it; 2 Department of Pharmacy, University of Naples Federico II, 80131 Naples, Italy; romina11provenzano@gmail.com (R.P.); ungaro@unina.it (F.U.); quaglia@unina.it (F.Q.); 3 Farmacia Florio, 80131 Naples, Italy; eflorio@farmaciaflorio.com; 4 Department of Molecular Medicine and Medical Biotechnology, University of Naples Federico II, 80131 Naples, Italy; chiara.pagliuca@unina.it (C.P.); giuseppe.mantova@unina.it (G.M.); elena.scaglione@unina.it (E.S.); mariateresa.vitiello2@unina.it (M.V.); roberta.colicchio@unina.it (R.C.); psalvato@unina.it (P.S.); 5 CEINGE, Advanced Biotechnologies s.c.ar.l., 80131 Naples, Italy; 6 Task Force on Microbiome Studies, University of Naples Federico II, 80131 Naples, Italy

**Keywords:** alcohol-based hand sanitizers, thickener agents, COVID-19

## Abstract

Hand hygiene, social distancing, and face covering are considered the first protection against Coronavirus spreading. The high demand during the COVID-19 emergency has driven a frenetic production and marketing of hand sanitizer gels. Nevertheless, the effect of the gelling agent and its amount on the effectiveness of alcohol-based hand sanitizers (ABHSs) needs to be clarified. We presented a systematic study on the effect of the characteristics and concentration of the most employed excipients on the properties and antimicrobial activity of ABHSs. Three different gelling agents, carbopol, hydroxypropylmethylcellulose (HPMC), and hydroxyethylcellulose (HEC), at four different concentrations were used to prepare ABHSs. Viscosity, spreadability, delivery from commercial dispensers, evaporation rate, rubbing time, and hand distribution of the ABHSs were then explored. Biocidal activity of selected ABHSs was evaluated in vitro on ATCC and clinical strains. The studied ABHS can be considered bioactive and comfortable. Nevertheless, the cellulose polymers and ethanol interactions led to a slight but significant reduction in the biocidal activity compared with carbopol-based formulations. Our results underline the importance of the gelling agent properties and support the choice of carbopol as one of the best thickener agents in ABHS formulations.

## 1. Introduction

Hand hygiene is considered one of the most effective strategies in preventing the transmission of microorganisms and virus infection across the public, healthcare workers, and from people to food [1,2], especially since January 2020, when the world was hit by the pandemic related to Coronavirus Disease-2019 (COVID-19). According to the authorities’ recommendations, hand hygiene, social distancing, and face covering have been considered the first protection against SARS coronavirus 2 (SARS-CoV-2) spreading during the last months. As reported by the World Health Organization (WHO), hand hygiene can be achieved by (i) handwashing with plain or antimicrobial soap and water, or by (ii) hand rubbing with waterless hand sanitizers [3]. Although handwashing is considered the first line of defense against spreading infection [4,5], hand sanitizers have showed significant virucidal activity against SARS-CoV-2 [6,7].

Hand sanitizers are available in different forms, i.e., liquids, gels, or foams, and they are applied on dry hands and rubbed over the fingers and hand surfaces until complete drying to kill transient bacteria and viruses. Between the hand sanitizers, alcohol-based hand sanitizers (ABHS) are the most employed and recommended by the authorities. The activity of ABHS is related to the ability of the alcohol (i.e., ethanol, iso-propanol, and n-propanol) to dissolve the lipid membranes and denature microbial proteins [8,9]. For this purpose, the most effective alcohol concentration is in the range 60–80% *v*/*v*, as suggested by the WHO and the Centre for Disease Control and Prevention (CDC). Alcohols show a non-specific, very broad spectrum and rapid germicidal activity [9,10,11,12,13], but no appreciable persistent effect is reported after their application. However, a reduction in the time of regrowth of skin bacteria is observed [14]. Although all the alcohols mentioned above appear adequate for ABHS production, ethanol is the most employed due to its compatibility with skin.

Due to the potential irritating effect on the skin related to the frequent use of ABHS, emollients and skin conditioners are usually required in their formulation. These excipients can reduce the alcohol drying effect on the skin and increase biocompatibility and user compliance [15,16,17,18]. Glycerin, a well-known humectant in pharmaceutical and cosmetic applications, is the most employed emollient in ABHS formulations. Glycerin increases skin hydration and interferes with the surface pH and superficial sebum content, although these alterations do not compromise the skin barrier function [16].

The ABHSs most employed and appreciated by users are formulated as gels, which can reduce the issues of handleability and risk of spillage of liquids and increase the alcohol evaporation rate, improving the antimicrobial effect [19,20]. Gels are obtained by incorporating a viscosity enhancer into the diluted alcohol. A wide variety of gelling agents approved for pharmaceutical and cosmetic products can be used for that purpose, but the most employed in ABHS formulations are cellulose derivates, such as Hydroxypropylmethylcellulose (HPMC) (e.g., Nutragel, NutraBe) and Hydroxyethylcellulose (HEC) (e.g., Hygienizing gel, Kaleido Studio), and carbopol (e.g., DermoGel, Glenova cosmetics) [16,21,22,23].

In the last months, with the COVID-19 pandemic the interest in hand ABHSs has hugely increased, as they are considered the gold standard for hand hygiene in health care. Although the WHO and CDC recommend frequent handwashing with soap and water as the first defense line to reduce the virus spread, numerous studies report the superior biocidal effect of ABHS on hand pathogen, especially for multi-drug resistant bacteria [24,25,26]. The high demand for ABHS during the first period of the emergency has driven cosmetic, pharmaceutical, and chemical companies into a frenetic delivery of products, which have fallen under different regulations to be marketed according to their composition and tested efficacy. Due to the massive request of hand sanitizers during the first months of the pandemic, Italian pharmacists have been directly involved in the ABHS production and dispensing. The galenic preparation of ABHS was suggested by SIFAP (Italian Society of Compounding Pharmacists) based on the monography “Dilute Ethanols” in the British Pharmacopoeia. This monography authorizes the pharmacists to prepare ethanol at an alcoholic grade lower than 96% (*v*/*v*) without any medical prescription [27]. To improve the handleability of the product, SIFAP recommended the addition of a gelling agent selected among cellulose derivates and carbopol [27].

According to the main premise that the effectiveness of ABHS is chiefly related to alcohol activity, the antimicrobial effect of the products remains unexplored. A few recent studies have focused on the influence of formulation composition on some ABHS properties (e.g., viscosity, handleability, rheological properties, and alcohol content) [21,23,28]. In contrast, the effect of gelling agent type and concentration on the effectiveness of alcohol-based hand sanitizers has not been clarified yet.

Here, we present a systematic study on the effect of the characteristics and concentration of the most employed excipients on the properties and antimicrobial activity of ABHSs. Three different gelling agents, Hydroxyethyl-cellulose (HEC), Hydroxypropylmethyl-cellulose (HPMC) and carbopol, at four different concentrations were used. Viscosity, rheological behavior, spreadability, delivery from commercial dispensers, solvent evaporation rate, rubbing time, and gel distribution on hands after application were explored. Finally, biocidal activity of selected ABHSs was evaluated in vitro following the bactericidal activity phase 2/step 1 and the fungicidal activity phase 2/step 1. Different ATCC and clinical bacterial strains and one fungal strain were exposed to the produced ABHSs to verify their activity spectrum.

## 2. Results

### 2.1. ABHS Characterization

The concentration of the gelling agent employed to produce gels was maintained below 2% to ensure its complete dissolution in the ethanol-rich water phase. In line with previous studies [21], gels were transparent at visual inspection and maintained transparency upon storage (transmittance was always higher than 70%, Figure S1).

**Figure 1:**
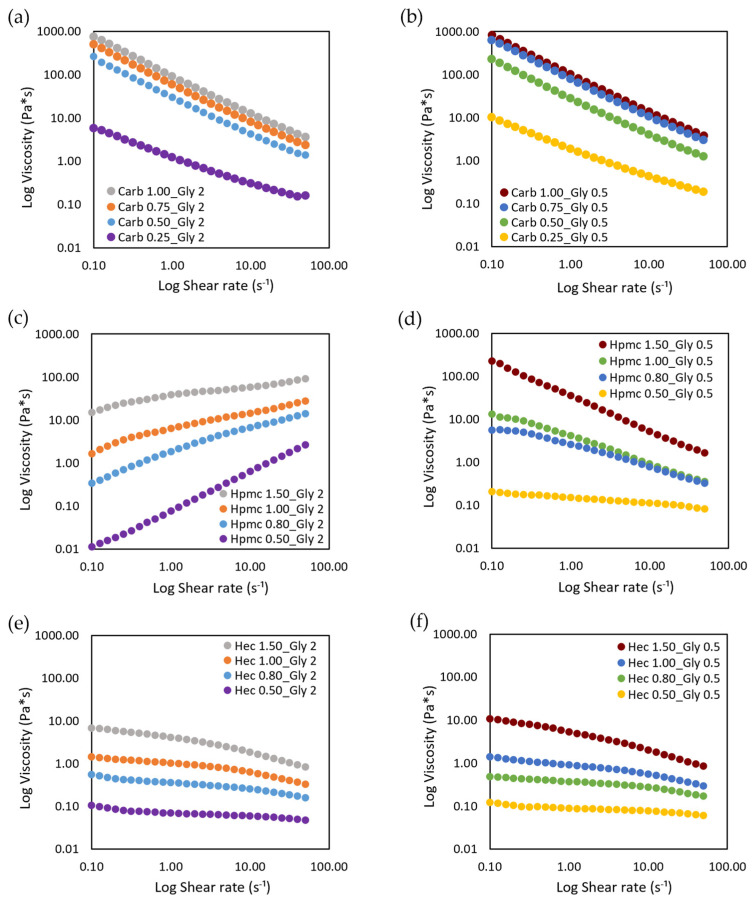
Viscosity vs. shear-rate curves of alcohol-based gel sanitizers. The curve is the average of three measures. (**a**,**b**) Carbopol-based gels; (**c**,**d**) HPMC-based gels; (**e**,**f**) HEC-based gels.

The effect of gel composition on shear viscosity was evaluated and the results of viscosity plotted against shear rate are reported in Figure 1.

The ABHS, as polymeric dispersions, displayed a typical shear-thinning behavior, in which the viscosity decreases and the shear stress increases by increasing the shear rate, independently of the thickener agent used (Figure S2, Supplementary Materials). In cellulose based ABHS, by decreasing the polymer concentrations and the viscosity the rheological behavior shifted from pseudoplastic to Newtonian (HPMC 0.50 and HEC 0.50). The decrease in shear viscosity was detected also at a low shear rate, thus no yield stress point was found in any tested ABHS. No differences were observed when increasing the amount of glycerin from 0.5% to 2%.

**Figure 2:**
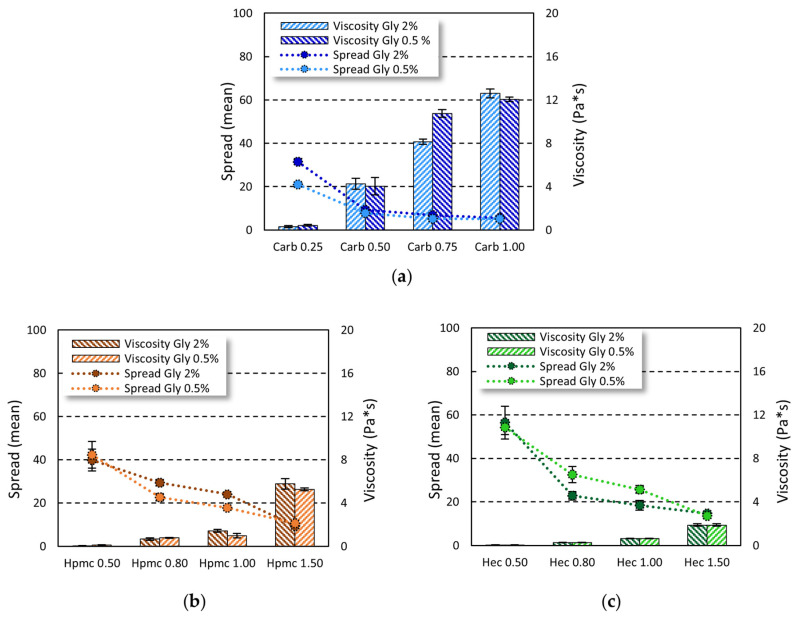
Viscosity and spreadability of alcohol-based hand sanitizers. (**a**) Carbopol-based ABHS; (**b**) HPMC-based ABHS; and (**c**) HEC-based ABHS.

On the other hand, the viscosity values were influenced by the gelling agent used (carbopol viscosity > HPMC viscosity > HEC viscosity) and its amount (lower viscosity at lower concentrations). To this purpose, the complex viscosity was measured and the effect of gel composition on the complex viscosity and spreadability was investigated (Figure 2).

By increasing the concentration of carbopol from 0.25% to 1.00%, viscosity increased 40-fold and 30-fold at 2% and 0.5% of glycerin, respectively. The same trend was observed in cellulose-based gels, where the increase in gelling agent from 0.5% to 1.5% led to a 90-fold and 45-fold increase in gel viscosity in Gly 2% and Gly 0.5% gels, respectively, for HPMC and a 30-fold increase in both Gly 2% and Gly 0.5% gels for HEC. On the other hand, by increasing the amount of gelling agent, a decrease in spreadability was observed. At the gelling agent concentrations tested, no representative changes in both viscosity and spreadability were seen when increasing the glycerin amount from 0.5% to 2%.

**Figure 3:**
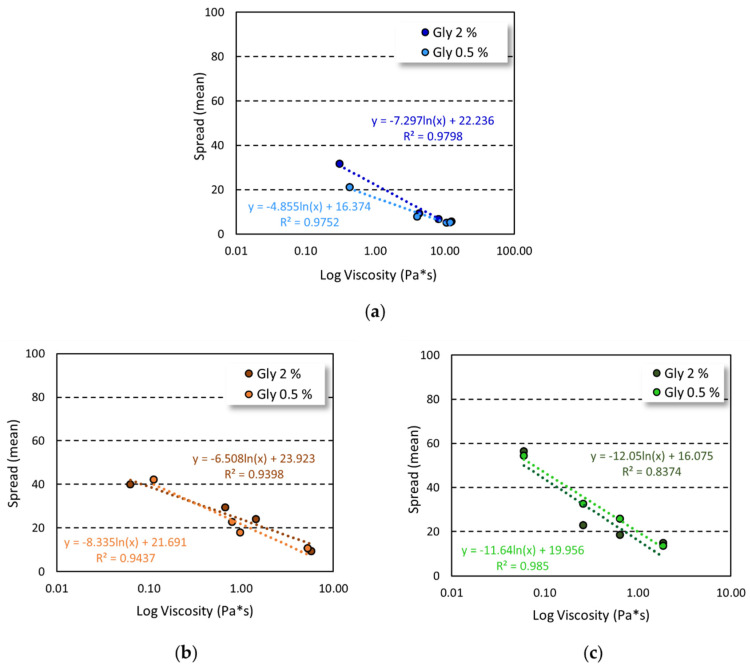
Relationship between viscosity and spreadability of the alcohol-based gels. Spreadability plotted against the viscosity of gel based on carbopol (**a**), HPMC (**b**), and HEC (**c**).

The increase in gelling agent concentration and viscosity of the formulation were closely related to spreadability, as demonstrated by the good correlation obtained by plotting spreading values and viscosity (Figure 3).

A good correlation was found for all the tested formulations with a R^2^ ≥ 0.9, except for HEC-based gels prepared employing 2% of glycerin, in which a R^2^ = 0.8374 was observed. Nevertheless, a plot with R^2^ ≥ 0.99 was achieved by considering the formulation in the highest range of viscosity values (Figure S3, Supplementary Materials).

### 2.2. Solvent Evaporation Rate

**Figure 4:**
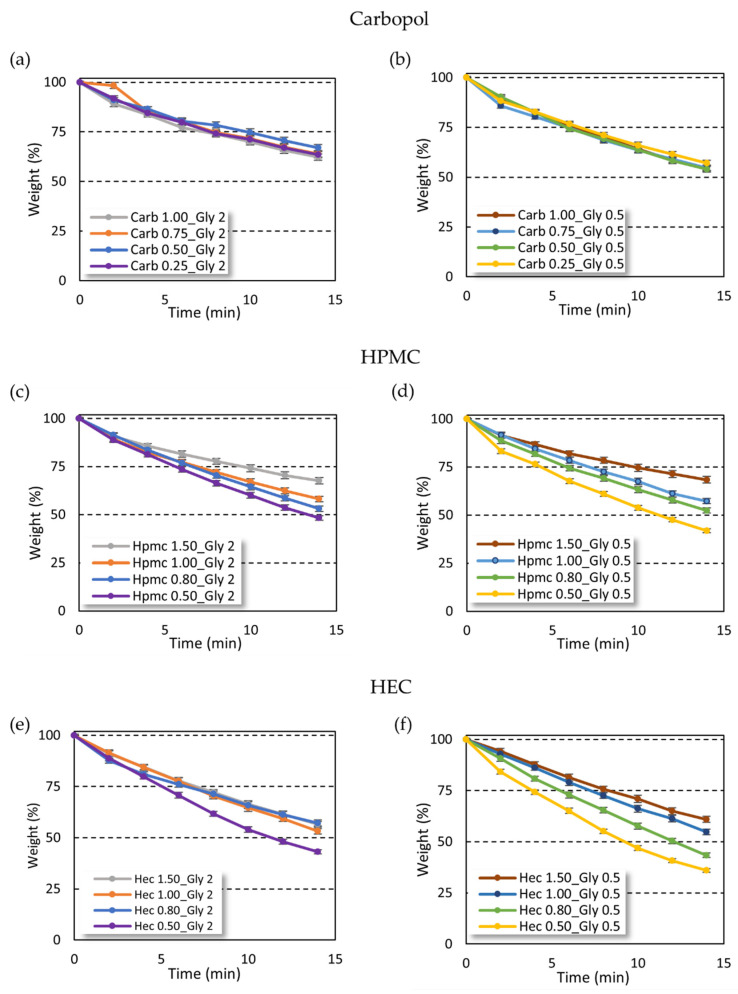
The hydro-alcoholic solvent evaporation rate from gels. The results are reported as weight of gel percentage in the time. (**a**,**b**) Carbopol-based gels; (**c**,**d**) HPMC-based gels; (**e**,**f**) HEC-based gels.

The weight loss of gel due to solvent evaporation from carbopol and cellulose-based gels was profoundly different (Figure 4). Interestingly, the solvent evaporation rate in carbopol-based gels was independent of gelling agent amount (Figure 4a,b), while an increase of the evaporation time was instead observed at increasing concentrations of the gelling agent in the cellulose gel series (Figure 4c,d,f), except for HEC-based gels at high gelling agent percentage (Figure 4e).

On the other hand, the increase in glycerin amount provided a reduction of solvent evaporation time only in gels with high viscosity. In particular, the effect of glycerin concentration was observed in all the carbopol-based gels at different gelling agent concentrations. In contrast, in the cellulose-based gels the effect of glycerin was found only in the samples with low gelling agent concentration and viscosity (Figure S4, Supplementary Materials). When increasing the gelling agent concentration, and thus the viscosity of the gels, no differences in the solvent evaporation rate were found.

The use of carbopol as a gelling agent led to an increase in hydro-alcoholic solvent evaporation time as compared with cellulose-based gels. The carbopol gels lost no more than 40% of the initial weight in 14 min, while both HEC- and HPMC-based gels showed a 50% loss of the initial weight after 14 min with the only exception for high gelling agent percentage (HEC 1.5% and HPMC 1.5%).

Interestingly, while the increase in carbopol amount did not influence the solvent evaporation rate, the cellulose-based gels showed reduced solvent evaporation time only at higher gelling agent percentage. This trend cannot be attributed to the higher viscosity of gels, which was also achieved in the carbopol series, but suggests an interaction between cellulose polymers and ethanol, such as hydrogen bonds and/or hydrophobic interactions [29].

### 2.3. ABHS Delivery

**Figure 5:**
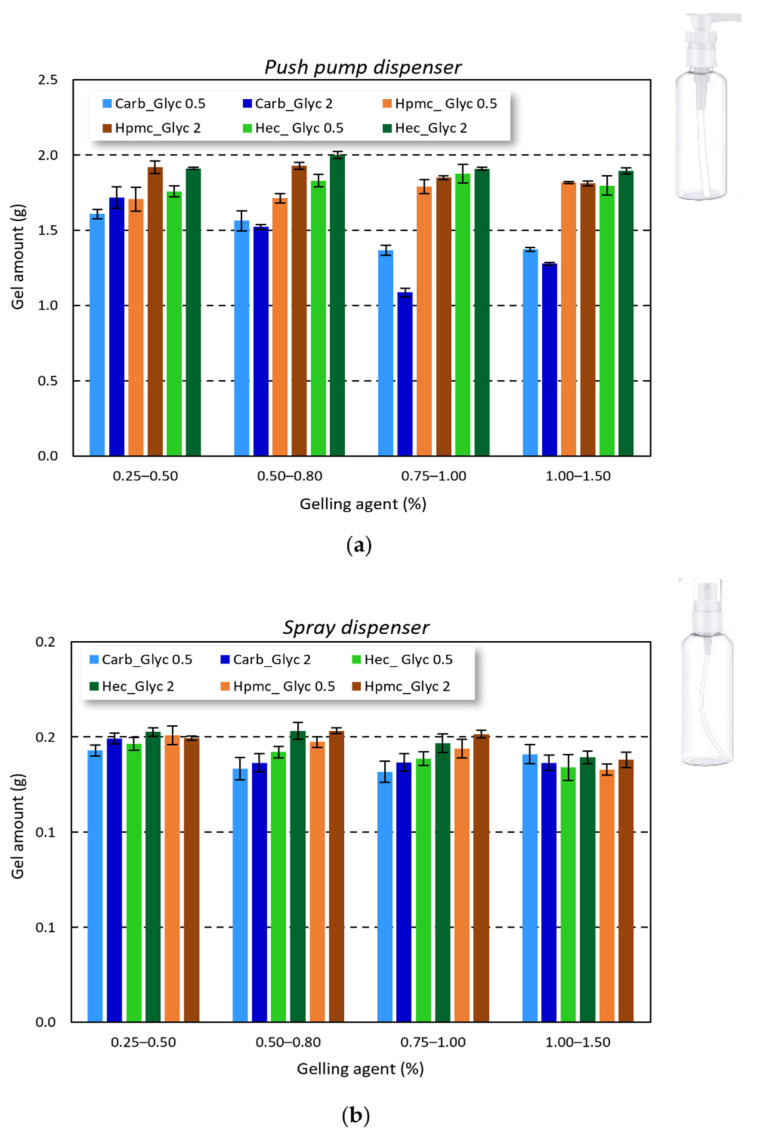
Gel amount delivered by the commercially available bottles: (**a**) push pump and (**b**) spray dispenser.

Considering the final gel application and the influence of gel amount applied to achieve an effective antimicrobial activity, the correct delivery and reproducibility of the gel amount delivered by the commercially available bottles is crucial. As reported in Figure 5, all the studied gels can be easily delivered by both push pump and spray dispenser. The delivered amount was very close for the cellulose-based gels (1.816 ± 0.083 g for HPMC-based gels and 1.872 ± 0.075 g for HEC-based gels), while for carbopol-based gels a slight reduction in the delivered amount was observed (around 1.705 ± 0.097 g). Nevertheless, the delivery of all the tested formulations can be considered reproducible.

### 2.4. ABHS Dry Time

**Figure 6:**
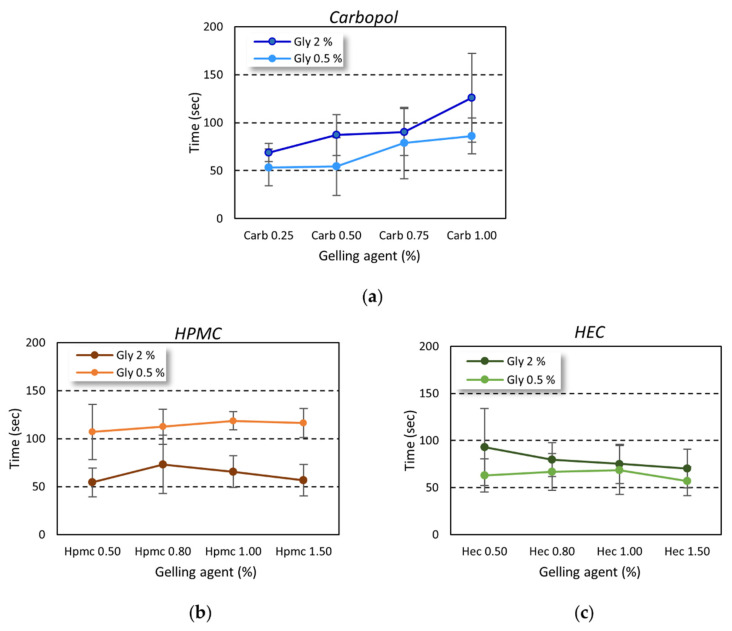
The gel dry time by rubbing. The results are reported as time needed to achieve complete drying by rubbing a known amount of gel on the hands of volunteers. (**a**) Carbopol-based gels; (**b**) HPMC-based gels; (**c**) HEC-based gels.

The gel dry time was evaluated, as described above, by monitoring the time needed to achieve a complete drying of a known gel amount applied on the hands of volunteers by rubbing. The trend was dependent on the gelling agent used (Figure 6). The rubbing time was always higher than 20–30 s, as reported by the WHO as the time needed to achieve correct hand hygiene [3].

While in carbopol-based gel an increase in the drying time was observed at increasing gelling agent percentage (Figure 6a), the drying time was independent of the amount of gelling agent employed in the cellulose-based series (Figure 6b,c).

### 2.5. ABHS Hand Distribution

**Figure 7:**
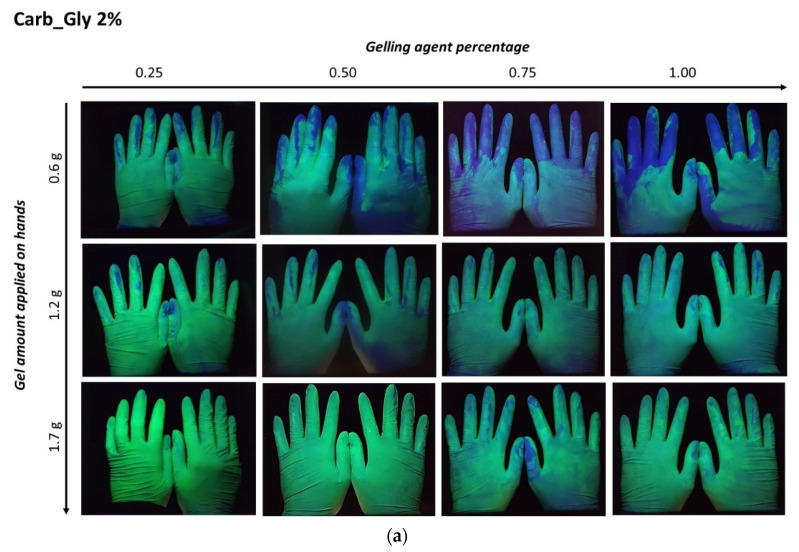

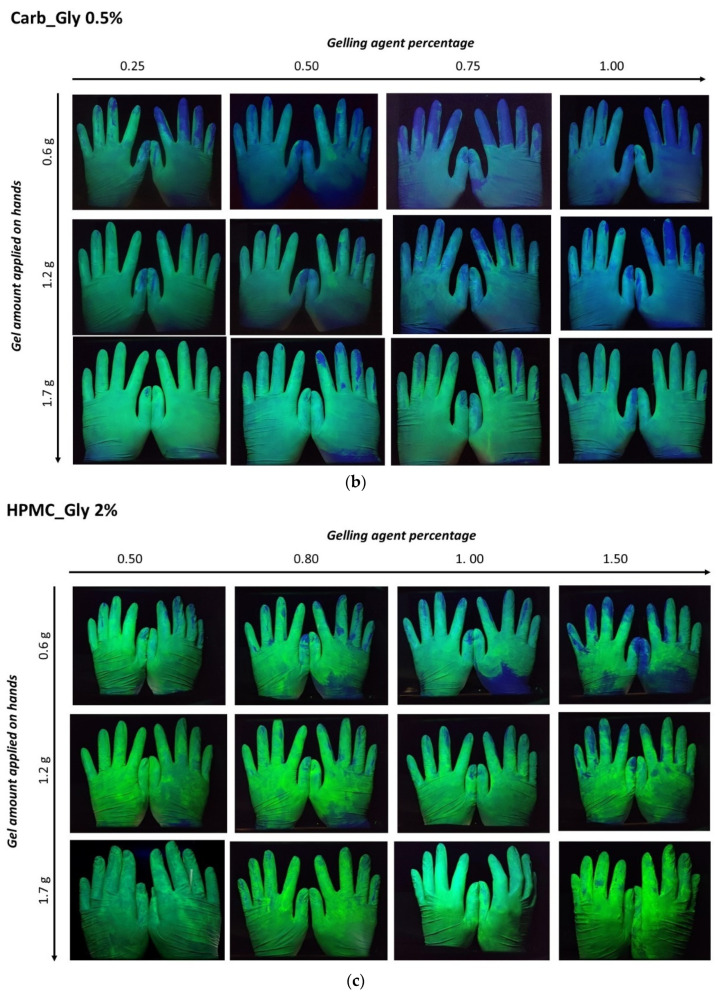

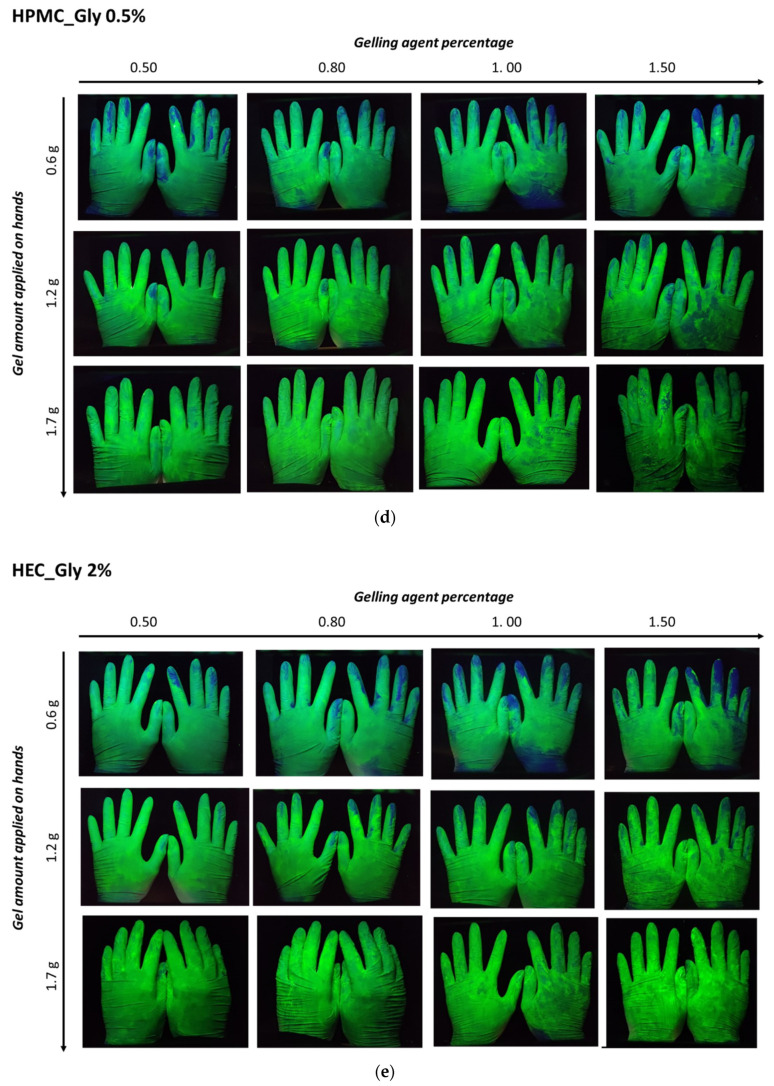

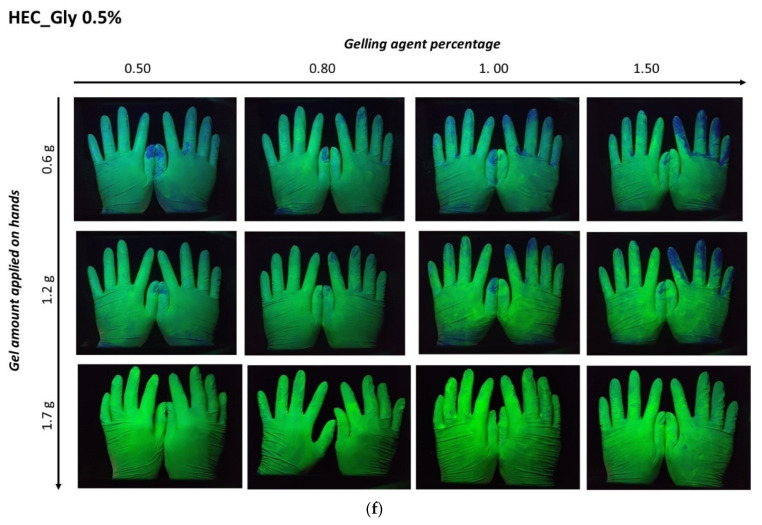
Distribution of fluorescent gels on hands after rubbing. (**a**) Carbopol-based gels containing 2% Glycerin (Carb_Gly 2%); (**b**) carbopol-based gels containing 0.5% Glycerin (Carb_Gly 0.5%); (**c**) HPMC-based gels containing 2% Glycerin (HPMC_Gly 2%); (**d**) HPMC-based gels containing 0.5% Glycerin (HPMC_Gly 0.5%); (**e**) HEC-based gels containing 2% Glycerin (HEC_Gly 2%); (**f**) HEC-based gels containing 0.5% Glycerin (HEC_Gly 0.5%). Representative images are shown.

The distribution of ABHS on the hands was evaluated by delivering a known amount of fluorescent gel. Three different amounts of fluorescent gel (1.7 g, 1.2 g, and 0.6 g) were placed on the palms of volunteers, and after rubbing and complete gel drying images of the hands were captured under a UV lamp (Figure 7).

For all the tested formulations, an effect of the gelling agent concentration and the gel amount applied was evident. In ABHS prepared employing high gelling agent concentrations (gels containing 1% of carbopol or 1.5% of cellulose polymers), a non-homogeneous distribution was observed. According to the higher viscosity and lower spreadability, the increase in the gelling agent percentage led to a less homogeneous gel distribution. Nevertheless, the effect of gel viscosity and spreadability was reduced by increasing the applied amount of ABHS (>1.0 g). In this case, in all the tested formulations the hand surface was completely covered.

### 2.6. Antimicrobial Activity

**Figure 8:**
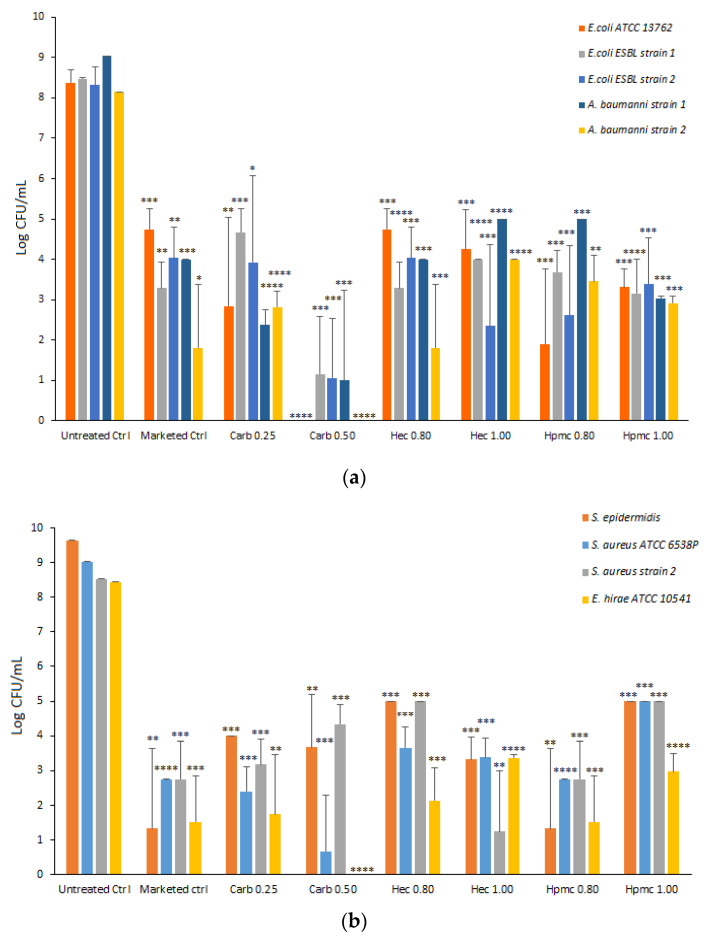
In vitro antimicrobial activity of alcohol-based gels against Gram-negative bacteria (**a**) and Gram-positive bacteria (**b**) (* *p* < 0.05, ** *p* < 0.01, *** *p* < 0.001 and **** *p* < 0.0001).

The antimicrobial activity of carbopol and cellulose-based gels, with glycerin at 2% (*w*/*w*), was evaluated against the selected test microorganisms (*E. coli*, *A. baumannii*, *S. epidermidis*, *S. aureus*, and *E. hirae*) (Figure 8).

A marketed gel sanitizer (Simply Gel, Geochemica, Italy) was also evaluated in parallel. Each gel formulation was mixed directly with bacterial suspension for one minute and the average CFU remaining was assessed by plating.

All the formulations tested significantly reduced the viability of each selected strain. As shown in Figure 8, the Carb0.5 formulation drastically reduced the viability of all Gram-negative strains tested with a reduction ≥ 7 Log CFU, while the other gels resulted in 5–6 Log CFU reduction (HPMC gels toward *E. coli* ATCC and *E. coli* ESBL strain 1, HEC gels for *E. coli* ESBL strain 2 and *A. baumanii* strain 2) (Figure 8). In the case of Gram-positive strains, the carbopol 0.5 gel dramatically reduced the viability of the *S. aureus* strain ATCC and *E. hirae* ATCC with 8 Log CFU reduction, whereas the HEC 1.0 formulation reduced the viability of *S. aureus* MRSA strain 2 with a decrease of 7 Log CFU and the HPMC 0.8 formulation that of *S. epidermidis* with a reduction of 8 Log CFU (Figure 8). Moreover, the gel formulations tested were significantly active against *C. albicans*, with the Carb 0.25 gel showing the highest antifungal activity (Figure S5, in the Supplementary Materials). The antimicrobial activity of an ethanol: water solution at 66% *w*/*w* (corresponding to 73% *v*/*v*) was tested as the control and no microorganism viability was observed in any tested strain (data not shown).

## 3. Discussion

The high request for hand sanitizer gels during the COVID-19 emergency has driven the frenetic marketing of products under different regulation frameworks. In fact, numerous alcohol-based gels have been placed in the EU market under cosmetic products legislation, which does not require any test proving the biocidal activity. In Europe, sanitizer gels need to be approved by the national competent authorities as biocidal products. At the same time, they are generally considered as over-the-counter products by the Food and Drug Administration (FDA) (https://www.fdabasics.com/fda-requirements-for-hand-sanitizers/, accessed on 27 January 2022). Alcohol-based hand sanitizers (ABHSs), especially when employing ethanol, are the most used and recommended by the authorities for correct and safe hand hygiene. To be effective as a biocide, the ethanol concentration in the gel must be between 60% and 85% [30]. As a paradox, ethanol percentages higher than 85% appear less effective. This effect is probably due to an ineffective protein denaturation in dry conditions and the rapid ethanol evaporation at high alcohol grade, which can reduce the contact time crucial for the biocide effect [30,31,32].

To understand the effect of the gelling agent used and its amount on the ethanol antimicrobial efficacy, we produced and characterized ABHS with ethanol concentration in the optimal alcohol grade range, i.e., 66.5% *w*/*w*, at two different concentrations (0.5% *w*/*w* and 2% *w*/*w*) and employing three gelling agents. HEC, HPMC, or carbopol were added to the ethanol: water mixture at four different concentrations in the range of 0.25–1.5% *w*/*w*. At the formulation condition used, all the gels were transparent and no cloudiness due to insoluble polymer particles was observed, suggesting that the polymers were homogeneously dispersed in the hydro-alcoholic solution, according to recently published studies [21].

The viscosity of the gels was reduced compared to aqueous gels prepared with the same final gelling agent concentration (data provided by manufacturers). The change in ethanol percentage provide a bell-shaped effect on gel viscosity, in which at lower alcoholic grade an increase in gel viscosity was achieved by increasing the ethanol concentration up to a maximum value, followed by a decrease of viscosity at alcoholic grade higher than 40–50% [21,33]. The effect of ethanol on gel viscosity has been related to the swelling of polymers in the hydro-alcoholic solvent. With increased alcohol concentration, the polymer hydration decreases, resulting in the loss of interchain interactions [34]. A similar effect has been already observed for carbopol-based formulations [35].

Considering the rheological properties, all the tested ABHS, as polymeric dispersions, displayed a typical shear-thinning behavior in which the viscosity decrease and the shear stress increase by increasing the shear rate (Figure 1, Figure S2). Nevertheless, a reduction in polymer concentration led to a shift from a pseudoplastic to Newtonian behavior, as detected in cellulose-based gels at lower gelling agent concentrations (HPMC 0.5 and HEC 0.5). As expected, the properties and the amount of the gelling agent influenced the ABHS and the gel spreadability. An increase in gel viscosity and a decrease in spreadability was achieved as the gelling agent percent increased. Nevertheless, all the tested gel formulations showed a consistency suitable for a comfortable hand application.

The spreadability of a semisolid formulation depends on three principal factors, i.e., surface tension, density, and viscosity, with the latter being the most crucial factor [36]. Thus, at the tested formulation conditions, the increase in gel viscosity led to a decrease in spreadability, providing a good correlation between complex viscosity and gel spreading. The correlation achieved (R^2^ ≥ 0.9), together with the absence of the yield stress, suggested that the viscosity was the main factor affecting the gel spreading [36], except for HEC-based gel prepared with 2% *w*/*w* of glycerin, in which the correlation was not maintained at high gelling agent concentration (Figure 3, Figure S3).

The gels were easily delivered by both a push pump and a nebulizer dispenser. The delivered amount appeared adequate to cover all hand surfaces. Nevertheless, the higher viscosity of carbopol-based gels was found to influence the deliverability. In particular, the delivery became difficult when increasing the gelling agent amount, suggesting that the carbopol concentration needs to be lower than 0.75% *w*/*w* to achieve handy formulations.

The distribution of ABHSs on hands after rubbing was also influenced by the gelling agent percentage employed. In particular, the increase of the gelling agent percentage, which increases the viscosity and reduces the spreadability, led to a reduction in homogeneous gel distribution (Figure 7). Nevertheless, the effect of gel viscosity and spreadability was decreased by increasing the applied amount of ABHS. In this case, the hand surface was completely covered for all the tested formulations.

The antimicrobial efficacy of ABHS was strictly related to ethanol interaction with the bacterial membrane. The additional components of ABHSs can improve ethanol effectiveness by limiting evaporation and increasing contact time with bacteria. The hydro-alcoholic solvent evaporation rate in carbopol-based formulations was independent by the gelling agent amount (Figure 4a,b), whereas it increased as the concentration of the gelling agent increased in the cellulose series (Figure 4c–f). These differences can be ascribed to the hydrogen bonds and/or hydrophobic interactions between the gelling agent (HPMC or HEC) and ethanol [29]. These interactions lead to a slight but significant reduction of the biocidal effect, which can be due to a lower amount of ethanol free to interact with the pathogen membrane. The carbopol-based gels, in which ethanol does not interact with polymer chains, showed a higher antimicrobial effect than cellulose-based formulations. This hypothesis was supported by the fact that ethanol: water mixture at 66.5% *w*/*w* (74% *v*/*v*) induced a 100% reduction of pathogen viability (data not shown), thus suggesting that ethanol–excipient interactions can strongly affect biocidal activity. Nevertheless, all the ABHSs showed a significant decrease in the viability of tested strains, and their antimicrobial effect was comparable or higher than that obtained by a marketed ABHS.

## 4. Conclusions

The huge request for gel hand sanitizers and the consolidated hypothesis that the use of ABHSs has become and will remain an integral part of everyday life, even when the COVID-19 pandemic is resolved, has prompted ABHS production. Although the sanitizer activity is exclusively attributed to the alcohol percentage (recommended to be at least 60% *v*/*v*), we highlighted that the gelling agent type and its concentration are both relevant in formulation handling and biocidal activity. We showed that the increase of gelling agent concentrations led to an increase in formulation viscosity and a decrease in spreadability. Nevertheless, differences in gelling agent interaction with ethanol were observed between ABHS prepared employing cellulose derivates (HEC and HPMC) and carbopol. Both HEC and HPMC showed a very similar behavior when dispersed in a hydro-alcoholic medium, and an increase in ethanol evaporation rate was observed by increasing the cellulose derivative amount. The interactions between the cellulose-derived polymers and ethanol led to a slight but significant reduction in the ABHS biocidal effect compared to carbopol-based formulations. The lower activity of cellulose-based ABHS can be ascribed to a lower fraction of ethanol ready to interact with the pathogen membrane. Our results underline the importance of the gelling agent–ethanol interactions in the formulation development and support carbopol as the most appropriate thickener agent in ABHS.

## 5. Materials and Methods

### 5.1. Materials

Carbopol 940 (40,000–60,000 mPa s, sol 0.5% 25 °C), Hydroxypropylmethylcellulose (HPMC) K15M (13,275–24,780 mPa s), Hydroxyethylcellulose (HEC) MR (4500–6500 mPa s 2%, 25 °C), and glycerin were purchased from Farmalabor (Italy). Triethanolamine (TEA) and fluorescein were purchased from Galeno (Italy) and Merck (UK), respectively. All salts and reagents were of analytical grade or better.

### 5.2. ABHS Production

The ABHSs were produced by adding HEC, HPMC, or carbopol as a gelling agent to a hydro-alcoholic solution with a final ethanol concentration of 66.5% *w*/*w*. Glycerin was added as a humectant at two different concentrations. The gel compositions are reported in Table 1.

Briefly, glycerin was added to diluted ethanol until complete dissolution. Soon after, the gelling agent was added to the mixture and homogeneously dispersed with a high shear lab mixer (Silverson L5M-A, Silverson Machines, Inc., East Longmeadow, MA, USA) at 8400 rpm for 10 min, up to homogeneous gel formation. When carbopol was used, gelation was achieved by adding triethanolamine (TEA) to the carbopol dispersion in water/ethanol. The obtained hydrogels were maintained at rest overnight before use. Fluorescently labelled gels were prepared by adding fluorescein to the hydro-alcoholic solution before adding the gelling agent, to achieve a final fluorescein concentration of 26.6 mg/100 g of gel.

### 5.3. ABHS Characterization

The viscosity and rheological behavior was measured using a rotational rheometer (Kinexus, Malvern, UK). First, the viscosity was evaluated at 25 °C, employing a CP4/40 cone-plate geometry, at a shear rate of 10 s^−1^. Results were reported as mean viscosity (Pa*s) ± standard deviation of three different measures. Flow curves of all samples were measured in the range of shear rate from 0.1 s^−1^ to 50 s^−1^. All the samples were analyzed in triplicate. The obtained results were reported as viscosity (Pa*s) vs. shear rate (s^−1^) plots.

The spreadability of ABHSs was assessed by a parallel-plate method [37]. Briefly, 0.5 g of the gel were placed in the center of a glass slide (20 × 20 cm), and carefully covered with another glass slide of the same dimension. A 0.5 g weight was placed on the covering glass slide for 5 min. The spread (S) of the formulation was calculated according to Equation (1):

(1) $$\;S=\frac{A2}{A1}\;$$

where A1 = the initial area (2 cm) and A2 = area after spreading. Results are reported as spread (ratio between area before and after spreading) ± standard deviation of three different measures.

The hydro-alcoholic medium evaporation rate from gels was evaluated by following their weight loss during the time. Briefly, 0.5 g of gel were applied on a microscope glass slide and maintained in a Petri dish at room temperature. At regular time intervals, the glass slides were weighted, and the weight loss was recorded. The results are reported as percent weight loss of the gel over time ± standard deviation of three different measures.

The ABHS amount delivered by commercial dispensers was evaluated. Two different bottles were used: (i) a bottle with a push pump dispenser (Cod: 011852, Farmalabor, Canosa Di Puglia, BT, Italy) and a bottle with a spray dispenser (Cod: 011321, Farmalabor, Canosa Di Puglia, BT, Italy). The bottles were filled with the gel and the delivered amount of formulation after dispenser activation was evaluated. The results are reported as the amount of the delivered gel (g) ± standard deviation of five different measures.

### 5.4. ABHS Rubbing Time

An amount of gel delivered by the push pump (approximately 1.70 g) described above (Cod: 011852, Farmalabor, Canosa Di Puglia, BT, Italy) was placed on the palms of volunteers. Ethical approval was not considered necessary for this study since gels were prepared by commercial excipients that fulfilled the quality requirements of Italian Pharmacopeia. The volunteers rubbed the gel onto the hands according to the WHO Guidelines on Hand Hygiene Technique with Alcohol-Based Formulation [3] until complete drying. The time interval from the start of rubbing to complete evaporation/absorption of the gel was recorded. After each test, volunteers washed their hands with soap before the following application. The results are reported as rubbing time until complete hand drying (min) ± standard deviation of five different measures.

### 5.5. ABHS Distribution on Hands

The distribution of ABHS on hands was evaluated using hydro-alcoholic gels containing a fluorescent label, the fluorescein. Three different amounts of fluorescently labelled gel (1.7 g, 1.2 g and 0.6 g), delivered by the push pump dispenser or by multiple spray dispenser activations, were placed on the hand palms of volunteers. The volunteers rubbed the gel onto the hands, according to the WHO Guidelines [3] as described above, until complete drying. The ABHS distribution was evaluated by hand irradiation in a black box type UV analyzer (Vilber Lourmat, Collégien, France), with a UV lamp at 245 nm (VL6-LC, Vilber Lourmat, Collégien, France). The volunteers used lactic gloves, and, after each test, they changed the gloves before the following application. Images of hands irradiated by the UV lamp were recorded.

### 5.6. Bacterial Strains and Growth Conditions

Bacterial strains employed in this study were: *Staphylococcus aureus* ATCC6538P (American Type Culture Collection, Manassas, VA, USA), *Escherichia coli* ATCC13762, *Enterococcus hirae* ATCC10541 and Multi Drug Resistant (MDR) clinical isolates of Meticillin Resistant *S. aureus* (MRSA), *S. epidermidis*, Extended Spectrum Beta Lactamase (ESBL)-producing *E. coli* (2 clinical strains), MDR *Acinetobacter baumannii* complex (2 clinical strains), and *Candida albicans*. The identification of clinical isolates was performed by matrix-assisted laser desorption/ionization (MALDI) mass spectrometer (Bruker Daltonics, Billerica, MA, USA) [38], and by biochemical phenotyping method in a VITEK^®^2 System (BioMérieux Italia S.p.a., Bagno A Ripoli (FI), Italy), according to the manufacturer’s instruction. The profile of susceptibility to antibiotics was also evaluated using the VITEK^®^2 System [39,40]. Bacterial isolates were aerobically cultured at 37 °C in Tryptic Soy (TS) broth/agar medium (Oxoid, Oxoid S.p.a., Rodano, Milan, Italy). Bacterial strains were stored frozen at −80 °C in TS broth with 10% glycerin (*v*/*v*) (Carlo Erba, Reagents, Milan, Italy).

### 5.7. In Vitro Antimicrobial Activity

The antimicrobial activity of the ABHS was evaluated against *S. aureus* ATCC6538P, *E. coli* ATCC13762, *E. hirae* ATCC10541, and against MDR clinical isolates of MRSA, *S. epidermidis*, ESBL -*E. coli*, MDR *A. baumannii* complex and *C. albicans*, taking a cue from EN 1500:2013. To prepare the working culture for each organism, a subculture from the stock culture was streaked onto TS Agar medium and incubated at 37 °C. After 18–24 h, a second subculture was prepared the same way and used to prepare the test suspension. A loopful of cells from the working culture was added in 5 mL of TS broth and incubated for 18–24 h. Then, 0.5 mL of this culture was inoculated in 100 mL of TS broth and incubated at 37 °C for 24 h, as indicated by EN 1500:2013 (Chemical disinfectants and antiseptics—Hygienic hand rub—test method and requirements (phase2/step2)). The test suspension was prepared by diluting the bacterial growth to 0.1 Optical density (O.D. 600 nm) in TS broth. A portion of 10 µL was added into each well of a 96-wells round-bottomed vinyl microplate (Costar, Corning incorporated, Corning, NY, USA) followed by 200 µL of ABHSs. After 60 s at room temperature, the whole content of each well was seeded on TS agar with 0.5 mL of Phosphate Buffer Saline (PBS) 1X. The plates were incubated overnight at 37 °C, and then viable cells were counted. All experiments were performed at least in triplicate. Results are reported as colony forming unit (CFU) ± standard deviation of at least three different replicates.

### 5.8. Statistical Analysis

Statistical analysis was performed by Student’s *t*-Test and * *p* < 0.05, ** *p* < 0.01, *** *p* < 0.001 and **** *p* < 0.0001 were utilized for statistical significance. All data are reported as mean value ± standard deviation (SD) calculated on at least triplicate experiments.

## Figures and Tables

**Table 1:** Composition of the ABHSs.

	**Carbopol**					
	**Formulation**	**Gelling** **Agent** **(% *w*/*w*)**	**TEA** **(% *w*/*w*)**	**Glycerin** **(% *w*/*w*)**	**Ethanol** **(% *w*/*w*)**	**Water** **(% *w*/*w*)**
Glycerin 2%	Carb 0.25_Gly 2	0.25	0.177	2.00	66.50	31.07
	Carb 0.50_Gly 2	0.50	0.384	2.00	66.50	30.62
	Carb 0.75_Gly 2	0.75	0.531	2.00	66.50	30.75
	Carb 1.00_Gly 2	1.00	0.811	2.00	66.50	29.69
Glycerin 0.5%	Carb 0.25_Gly 0.5	0.25	0.177	0.50	66.50	32.75
	Carb 0.50_Gly 0.5	0.5	0.384	0.50	66.50	32.50
	Carb 0.75_Gly 0.5	0.75	0.531	0.50	66.50	32.25
	Carb 1.00_Gly 0.5	1.00	0.811	0.50	66.50	32.00
	**Hydroxyethylcellulose—HEC**					
	**Formulation**	**Gelling** **Agent** **(% *w*/*w*)**	**Glycerin** **(% *w*/*w*)**	**Ethanol** **(% *w*/*w*)**	**Water** **(% *w*/*w*)**	
Glycerin 2%	Hec 0.50_Gly 2	0.50	2.00	66.50	31.07	
	Hec 0.80_Gly 2	0.80	2.00	66.50	30.70	
	Hec 1.00_Gly 2	1.00	2.00	66.50	30.50	
	Hec 1.50_Gly 2	1.50	2.00	66.50	30.00	
Glycerin 0.5%	Hec 0.50_Gly 0.5	0.50	0.50	66.50	32. 50	
	Hec 0.80_Gly 0.5	0.80	0.50	66.50	32.20	
	Hec 1.00_Gly 0.5	1.00	0.50	66.50	32.00	
	Hec 1.50_Gly 0.5	1.50	0.50	66.50	31.50	
	**Hydroxypropylmethylcellulose—HPMC**					
	**Formulation**	**Gelling** **Agent** **(% *w*/*w*)**	**Glycerin** **(% *w*/*w*)**	**Ethanol** **(% *w*/*w*)**	**Water** **(% *w*/*w*)**	
Glycerin 2%	Hpmc 0.50_Gly 2	0.50	2.00	66.50	31.00	
	Hpmc 0.80_Gly 2	0.80	2.00	66.50	30.70	
	Hpmc 1.00_Gly 2	1.00	2.00	66.50	30.50	
	Hpmc 1.50_Gly 2	1.50	2.00	66.50	30.00	
Glycerin 0.5%	Hpmc 0.50_Gly 0.5	0.50	0.50	66.50	32. 50	
	Hpmc 0.80_Gly 0.5	0.80	0.50	66.50	32.20	
	Hpmc 1.00_Gly 0.5	1.00	0.50	66.50	32.00	
	Hpmc 1.50_Gly 0.5	1.50	0.50	66.50	31.50	

## Data Availability

Data is contained within the article or supplementary material.

## Supplementary Materials

The following are available online at https://www.mdpi.com/article/10.3390/gels8020087/s1, Figure S1: Transmittance of cellulose-based gels. Samples transmittance was measured spectrophotometrically at 600 nm, using quartz cuvettes. Figure S2: Shear stress vs. shear-rate curves of carbopol, HPMC-and HEC-based alcohol-based gel sanitizers. The curve is the average of three measures. Figure S3: Relationship between viscosity and spreadability of the HEC-based gels prepared employing 2% of glycerin at the gelling agent concentration of 0.5%, 0.8% and 1.0%. Figure S4: Solvent evaporation rate from gels. Figure S5: In vitro antimicrobial activity of alcohol-based gels against Candida albicans clinical strain.

## References

[1] World Health Organization (2004). World Alliance for Patient Safety: Forward Programme, 2005.

[2] Lopez A.D., Mathers C.D., Ezzati M., Jamison D.T., Murray C.J. (2006). Global and Regional Burden of Disease and Risk Factors, 2001: Systematic Analysis of Population Health Data. Lancet.

[3] World Health Organization (WHO) (2009). WHO Guidelines on Hand Hygiene in Health Care: First Global Patient Safety Challenge Clean Care Is Safer Care.

[4] Kac G., Podglajen I., Gueneret M., Vaupré S., Bissery A., Meyer G. (2005). Microbiological Evaluation of Two Hand Hygiene Procedures Achieved by Healthcare Workers during Routine Patient Care: A Randomized Study. J. Hosp. Infect..

[5] Golin A.P., Bhsc D.C., Ghahary A. (2020). Hand Sanitizers: A Review of Ingredients, Mechanisms of Action, Modes of Delivery, and Efficacy against Coronaviruses. Am. J. Infect. Control.

[6] Chojnacki M., Dobrotka C., Osborn R., Johnson W., Young M., Meyer B., Laskey E., Wozniak R.A.F., Dewhurst S., Dunman P.M. (2021). Evaluating the Antimicrobial Properties of Commercial Hand Sanitizers. mSphere.

[7] Leslie R.A., Zhou S.S., Macinga D.R. (2021). Inactivation of SARS-CoV-2 by Commercially Available Alcohol-Based Hand Sanitizers. Am. J. Infect. Control.

[8] Gold N.A., Mirza T.M., Avva U. (2021). Alcohol Sanitizer.

[9] Larson E., Morton H., Block S.S. (1991). Alcohols. Disinfection, Sterilization and Preservation.

[10] Sakuragi T., Yanagisawa K., Dan K. (1995). Bactericidal Activity of Skin Disinfectants on Methicillin-Resistant Staphylococcus Aweus. Anesth. Analg..

[11] Kampf G., Jarosch R., Rüden H. (1998). Limited Effectiveness of Chlorhexidine Based Hand Disinfectants against Methicillin-Resistant Staphylococcus Aureus (MRSA). J. Hosp. Infect..

[12] Kampf G., Höfer M., Wendt C. (1999). Efficacy of Hand Disinfectants against Vancomycin-Resistant Enterococci In Vitro. J. Hosp. Infect..

[13] Rotter M. (2001). Arguments for Alcoholic Hand Disinfection*. J. Hosp. Infect..

[14] Gaonkar T.A., Geraldo I., Caraos L., Modak S.M. (2005). An Alcohol Hand Rub Containing a Synergistic Combination of an Emollient and Preservatives: Prolonged Activity against Transient Pathogens. J. Hosp. Infect..

[15] Filipe H.A.L., Fiuza S.M., Henriques C.A., Antunes F.E. (2021). Antiviral and Antibacterial Activity of Hand Sanitizer and Surface Disinfectant Formulations. Int. J. Pharm..

[16] Ahmed-Lecheheb D., Cunat L., Hartemann P., Hautemanire A. (2012). Prospective Observational Study to Assess Hand Skin Condition after Application of Alcohol-Based Hand Rub Solutions. Am. J. Infect. Control.

[17] Harbarth S., Pittet D., Grady L., Zawacki A., Potter-Bynoe G., Samore M.H., Goldmann D.A. (2002). Interventional Study to Evaluate the Impact of an Alcohol-Based Hand Gel in Improving Hand Hygiene Compliance. Pediatr. Infect. Dis. J..

[18] Kramer A., Bernig T., Kampf G. (2002). Clinical Double-Blind Trial on the Dermal Tolerance and User Acceptability of Six Alcohol-Based Hand Disinfectants for Hygienic Hand Disinfection. J. Hosp. Infect..

[19] Greenaway R.E., Ormandy K., Fellows C., Hollowood T. (2018). Impact of Hand Sanitizer Format (Gel/Foam/Liquid) and Dose Amount on Its Sensory Properties and Acceptability for Improving Hand Hygiene Compliance. J. Hosp. Infect..

[20] Fu L., Le T., Liu Z., Wang L., Guo H., Yang J., Chen Q., Hu J. (2020). Different Efficacies of Common Disinfection Methods against Candida Auris and Other Candida Species. J. Infect. Public Health.

[21] Perinelli D.R., Berardi A., Bisharat L., Cambriani A., Ganzetti R., Bonacucina G., Cespi M., Palmieri G.F. (2021). Rheological Properties of Cellulosic Thickeners in Hydro-Alcoholic Media: The Science behind the Formulation of Hand Sanitizer Gels. Int. J. Pharm..

[22] Fallica F., Leonardi C., Toscano V., Santonocito D., Leonardi P., Puglia C. (2021). Assessment of Alcohol-Based Hand Sanitizers for Long-Term Use, Formulated with Addition of Natural Ingredients in Comparison to Who Formulation 1. Pharmaceutics.

[23] Berardi A., Perinelli D.R., Merchant H.A., Bisharat L., Basheti I.A., Bonacucina G., Cespi M., Palmieri G.F. (2020). Hand Sanitisers amid COVID-19: A Critical Review of Alcohol-Based Products on the Market and Formulation Approaches to Respond to Increasing Demand. Int. J. Pharm..

[24] Casewell M., Law M., Desai N. (1988). A Laboratory Model for Testing Agents for Hygienic Hand Disinfection: Handwashing and Chlorhexidine for the Removal of Klebsiella. J. Hosp. Infect..

[25] Huang Y., Oie S., Kamiya A. (1994). Comparative Effectiveness of Hand-Cleansing Agents for Removing Methicillin-Resistant Staphylococcus Aureus from Experimentally Contaminated Fingertips. Am. J. Infect. Control.

[26] Wade J., Desai N., Casewell M. (1991). Hygienic Hand Disinfection for the Removal of Epidemic Vancomycin Resistant Enterococcus Faecium and Gentamicin-Resistant Enterobacter Cloacae. J. Hosp. Infect..

[27] SIFAP Etanolo Diluito, Formulazioni Farmacopea Britannica. https://www.sifap.org/newsletter/etanolo-bp,-formulazioni-farmacopea-britannica.

[28] Pasquini C., Hespanhol M.C., Cruz K.A.M.L., Pereira A.F. (2020). Monitoring the Quality of Ethanol-Based Hand Sanitizers by Low-Cost near- Infrared Spectroscopy. Microchem. J..

[29] Lindman B., Medronho B., Alves L., Costa C., Edlund H., Norgren M. (2017). The Relevance of Structural Features of Cellulose and Its Interactions to Dissolution, Regeneration, Gelation and Plasticization Phenomena. Phys. Chem. Chem. Phys..

[30] Edmonds S.L., MacInga D.R., Mays-Suko P., Duley C., Rutter J., Jarvis W.R., Arbogast J.W. (2012). Comparative Efficacy of Commercially Available Alcohol-Based Hand Rubs and World Health Organization-Recommended Hand Rubs: Formulation Matters. Am. J. Infect. Control.

[31] Suchomel M., Kundi M., Pittet D., Weinlich M., Rotter M.L. (2012). Testing of the World Health Organization Recommended Formulations in Their Application as Hygienic Hand Rubs and Proposals for Increased Efficacy. Am. J. Infect. Control.

[32] Wilkinson M.A.C., Ormandy K., Bradley C.R., Fraise A.P., Hines J. (2017). Dose Considerations for Alcohol-Based Hand Rubs. J. Hosp. Infect..

[33] Brown A.F., Jones D.S., Woolfson A.D. (1998). The Effect of Alcoholic Solvents on the Rheological Properties of Gels Composed of Cellulose Derivatives. J. Pharm. Pharmacol..

[34] Gupta P., Garg S. (2002). Recent Advances in Semisolid Dosage Forms for Dermatological Application. Pharm. Technol..

[35] Fresno M.J.C., Ramírez A.D., Jiménez M.M. (2002). Systematic Study of the Flow Behaviour and Mechanical Properties of Carbopol^®^ Ultrez^TM^ 10 Hydroalcoholic Gels. Eur. J. Pharm. Biopharm..

[36] Douguet M., Picard C., Savary G., Merlaud F., Loubat-bouleuc N., Grisel M. (2017). Spreading Properties of Cosmetic Emollients Use of Synthetic Skin Surface to Elucidate Structural Effect. Colloids Surf. B Biointerfaces.

[37] Garg A., Aggarwal D., Garg S., Singla A.K. (2002). Spreading of Semisolid Formulations: An Update. Pharm. Technol. N. Am..

[38] Sogawa K., Watanabe M., Sato K., Segawa S., Ishii C., Akiko M., Murata S., Saito T., Nomura F. (2011). Use of the MALDI BioTyper System with MALDI–TOF Massspectrometry for Rapid Identification of Microorganisms. Anal. Bioanal. Chem..

[39] Di Natale F., Manna L., La Motta F., Colicchio R., Scaglione E., Pagliuca C., Salvatore P. (2018). Capture of Bacterial Bioaerosol with a Wet Electrostatic Scrubber. J. Electrost..

[40] Colicchio R., Nigro E., Colavita I., Pagliuca C., di Maro S., Tomassi S., Scaglione E., Carbone F., Carriero M.V., Matarese G. (2021). A Novel Smaller Β-defensin-derived Peptide Is Active against Multidrug-resistant Bacterial Strains. FASEB J..

