# Supplementary material for: Alcohol-Based Hand Sanitizers: Does Gelling Agent Really Matter?
Source: Gels. 2022 Jan 29;8(2):87. doi: 10.3390/gels8020087 (PMC8871833; doi:10.3390/gels8020087)
Supplement: Supplementary file 1 [file gels-08-00087-s001.zip › gels-1524934-supplementary.pdf]

Alcohol-Based Hand Sanitizers: Does Gelling Agent Really Matter?

Ivana d'Angelo [1], Romina Provenzano [2], Ettore Florio [3], Chiara Pagliuca [4], Giuseppe Mantova [4], Elena Scaglione [4], Mariateresa Vitiello [4], Roberta Colicchio [4], Paola Salvatore [4, 5, 6], Francesca Ungaro [2], Fabiana Quaglia[2] and Agnese Miro [2,*]

[1] Department of Environmental, Biological and Pharmaceutical Sciences and Technologies, University of Campania "Luigi Vanvitelli", 81100 Caserta, Italy; ivana.dangelo@unicampania.it (I.d.)

[2] Department of Pharmacy, University of Naples Federico II, 80131 Naples, Italy; romina11provenzano@gmail.com (R.P.); ungaro@unina.it (F.U.); quaglia@unina.it (F.Q.); miro@unina.it (A.M)

[3] Farmacia Florio, 80131 Naples, Italy; eflorio@farmaciaflorio.com (E.F.)

[4] Department of Molecular Medicine and Medical Biotechnology, University of Naples Federico II, 80131 Naples, Italy; chiara.pagliuca@unina.it (C.P.); giuseppe.mantova@unina.it (G.M.); elena.scaglione@unina.it (E.S.); mariateresa.vitiello2@unina.it (M.V.); roberta.colicchio@unina.it (R.C.); psalvato@unina.it (P.S)

[5] CEINGE, Advanced Biotechnologies s.c.ar.l., 80131 Naples, Italy

[6] Task Force on Microbiome Studies, University of Naples Federico II, 80131 Naples, Italy

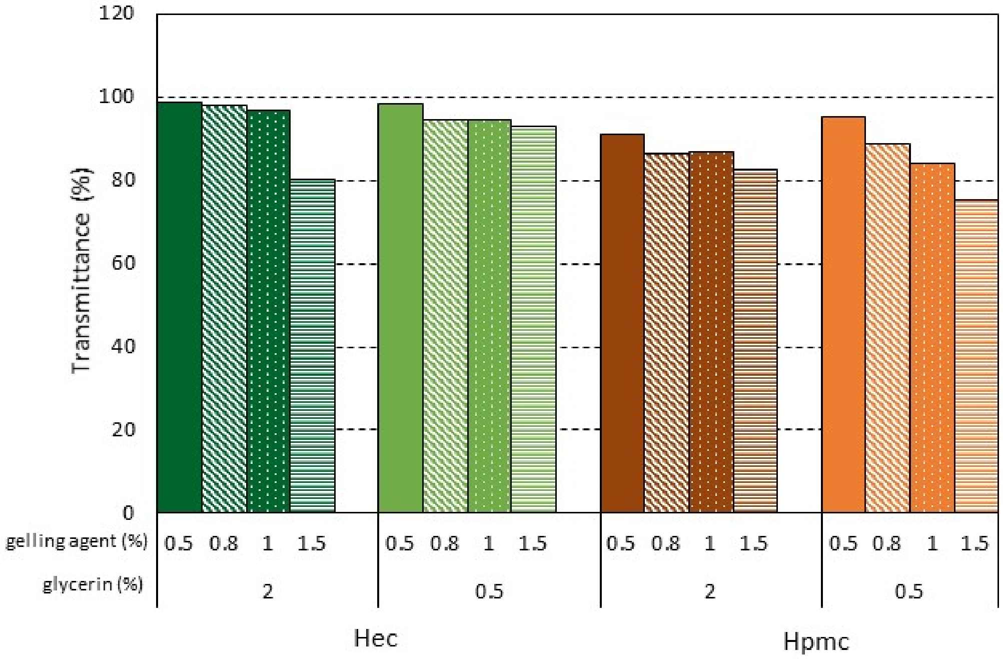

Figure S1: Transmittance of cellulose-based gels. Samples transmittance was measured spectrophotometrically (UV- 1800, UV Spectrophotometer, Shimad-zu, Japan) at 600 nm, using quartz cuvettes. Results are reported as a relative per-centage (%) as compared to pure water. All samples were analysed in triplicate.

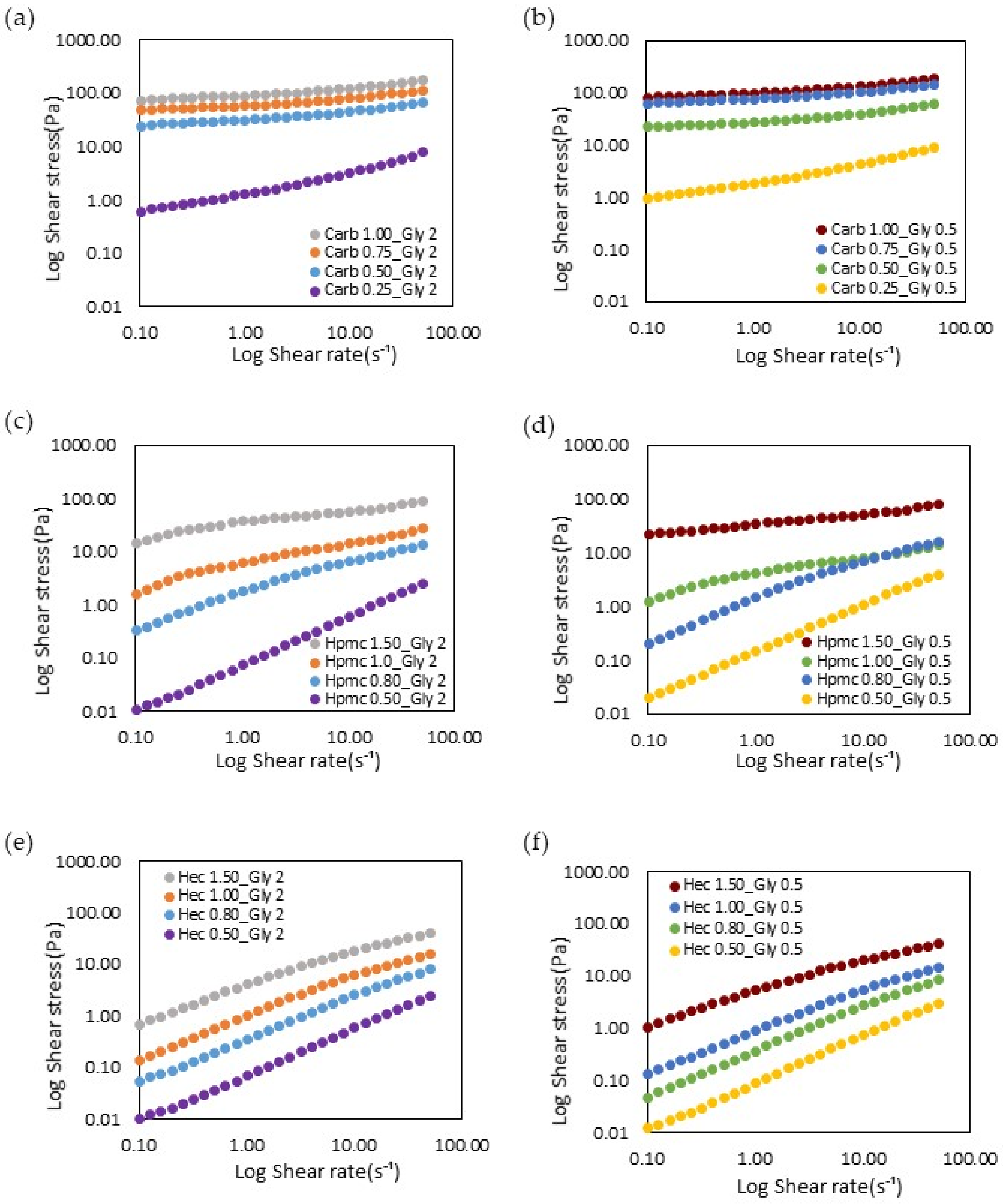

Figure S2: Shear stress vs shear-rate curves of alcohol based gel sanitizers. The curve is the average of three measures. (**a**,**b**) Carbopol-based gels; (**c**,**d**) HPMC-based gels; (**e**,**f**) HEC-based gels.

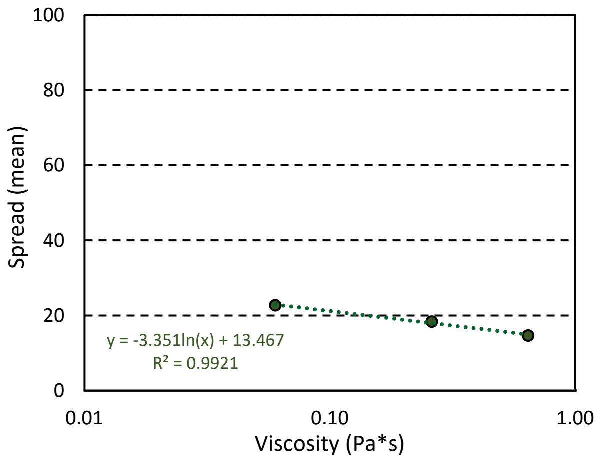

Figure S3: Relationship between viscosity and spreadability of the HEC-based gels prepared employing 2% of glycerin at the gelling agent concentration of 0.8 %, 1.0% and 1.5%

Carbopol

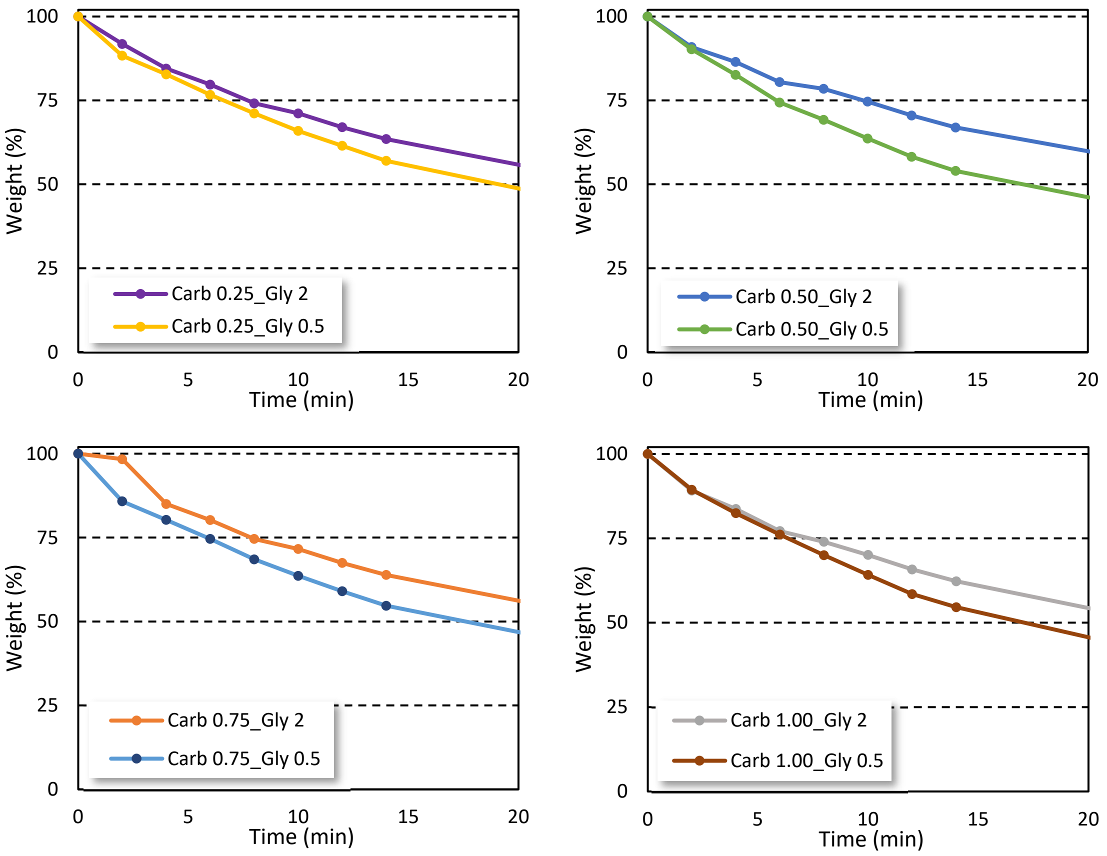

HPMC

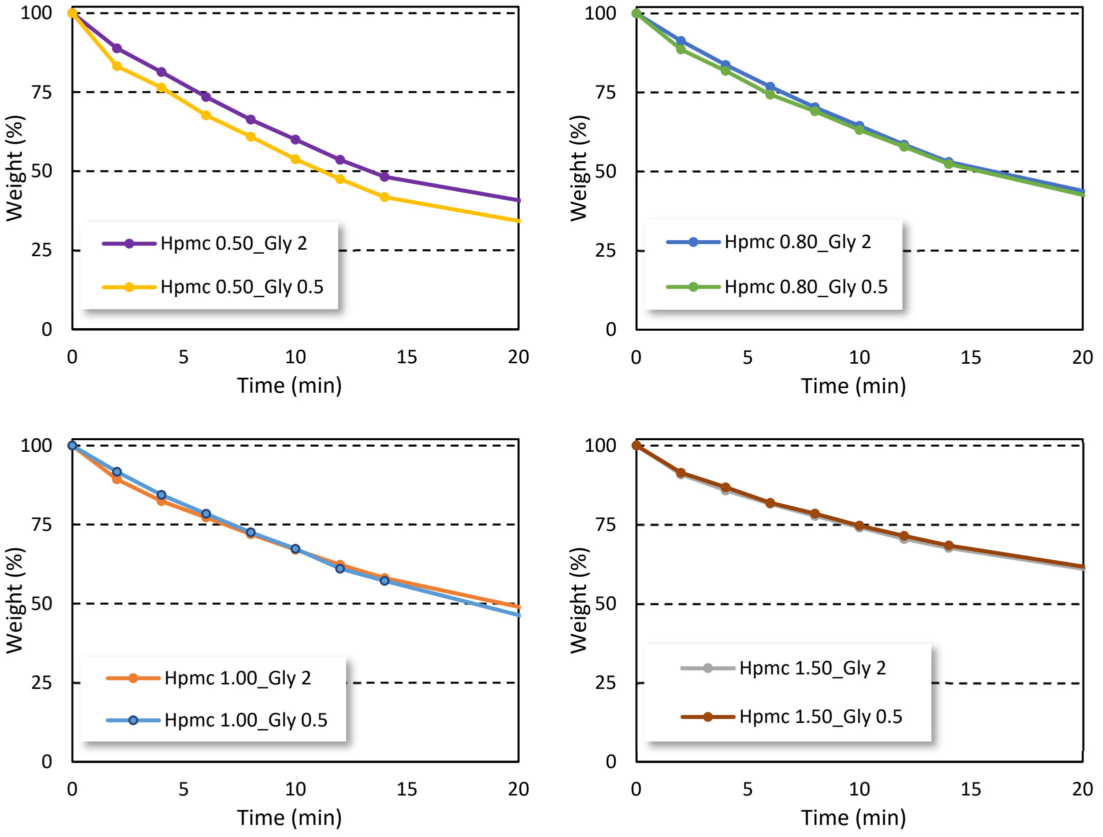

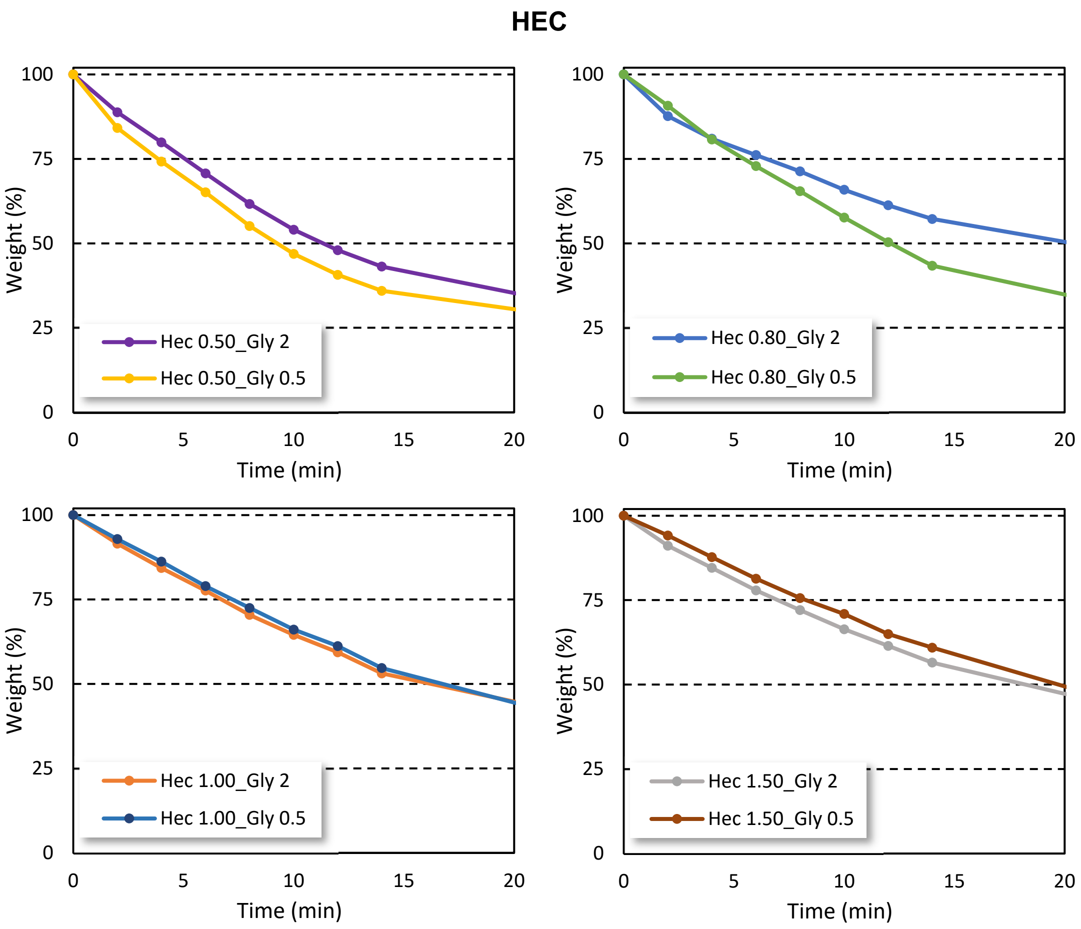

Figure S4: The hydro-alcoholic solvent evaporation rate from gels. The results are reported as weight of gel percentage in the time.

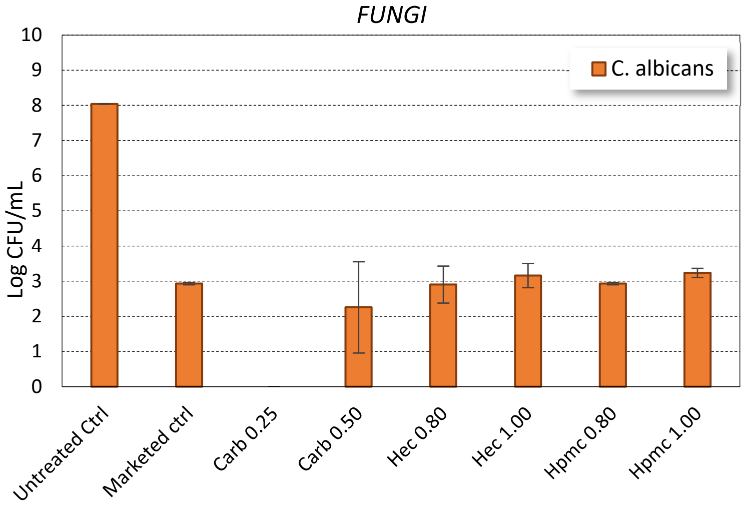

Figure S5: In vitro antimicrobial activity of alcohol-based gels against *Candida albicans* clinical strain.
